# Risk Factors Associated with Ischemic Stroke in the Immediate Postoperative Period of Cardiac Surgery

**DOI:** 10.21470/1678-9741-2022-0072

**Published:** 2023-06-14

**Authors:** Luana Quintana Marchesan, Marco Aurélio Lumertz Saffi, Leticia Fioravante da Silveira, Maria Clara Marramarco Lovato, Pedro Cargnelutti de Araujo, Diego Chemello

**Affiliations:** 1 Postgraduate Program in Health Science, Universidade Federal de Santa Maria (UFSM), Santa Maria, Rio Grande do Sul, Brazil; 2 Department of Cardiology, Hospital de Clínicas de Porto Alegre (HCPA), Porto Alegre, Rio Grande do Sul, Brazil; 3 Department of Clinical Medicine, Hospital Universitário de Santa Maria (HUSM), Santa Maria, Rio Grande do Sul, Brazil; 4 Department of Clinical Medicine, Centro de Ciências da Saúde, Universidade Federal de Santa Maria (UFSM), Santa Maria, Rio Grande do Sul, Brazil

**Keywords:** Stroke, Risk Factors, Thoracic Surgery, Cohort Studies, Postoperative Complications

## Abstract

**Introduction:**

Stroke remains a major complication of cardiac surgery. Despite all efforts, the incidence of postoperative stroke remains as high as 6%. We aimed to investigate risk factors for ischemic stroke in a contemporary cohort of patients undergoing cardiac surgery.

**Methods:**

This is a retrospective cohort study of 678 consecutive adult patients who underwent cardiac surgery requiring cardiopulmonary bypass in a tertiary hospital in Brazil between July 1, 2011, and December 31, 2018. The primary outcome was the rate of early (perioperative and seven-day postoperative) stroke, defined as the occurrence of the outcome during the index admission. We developed a predictive model of stroke using the Poisson regression analysis with robust variance.

**Results:**

Postoperative stroke occurred in 24 patients (3.5%), 23 (3.3%) were ischemic, and 21 (3.0%) were diagnosed in the first 72 hours after surgical procedure. After multivariate analysis, the following factors were significantly associated with stroke: previous stroke/transient ischemic attack (relative risk [RR]=2.75; 95% confidence interval [CI], 1.11-6.82), carotid artery disease (RR=4.0; 95% CI, 1.43-11.0), previous atrial fibrillation (RR=3.26, 95% CI, 1.31-8.1), and postoperative platelets > 200,000/mm^3^ (RR=2.26; 95% CI, 1.01-5.1).

**Conclusion:**

We developed a contemporary model to determine risk predictors for stroke after cardiac surgery. This model may help clinicians to identify patients at risk and could be useful in clinical practice.

**Table:** 

Abbreviations, Acronyms & Symbols				
AF	= Atrial fibrillation		EuroSCORE	= European System for Cardiac Operative Risk Evaluation
AVR	= Aortic valve replacement		ICU	= Intensive care unit
CABG	= Coronary artery bypass grafting		INR	= International normalized ratio
CAD	= Coronary artery disease		IQR	= Interquartile range
CAS	= Carotid artery stenosis		MI	= Myocardial infarction
CHF	= Congestive heart disease		MRI	= Magnetic resonance imaging
CI	= Confidence interval		MRS	= Myocardial revascularization surgery
COPD	= Chronic obstructive pulmonary disease		MVR	= Mitral valve replacement
CPB	= Cardiopulmonary bypass		PAD	= Peripheral arterial disease
CRP	= Cardiorespiratory arrest		POAF	= Postoperative atrial fibrillation
CT	= Computed tomography		RR	= Relative risk
DIVC	= Disseminated intravascular coagulation		SAH	= Systemic arterial hypertension
DM	= Diabetes mellitus		TIA	= Transient ischemic attack
ECC	= Extracorporeal circulation			

## INTRODUCTION

Each year, approximately 795,000 people experience a new or recurrent stroke in the United States of America. Approximately 610,000 of these are first attacks. Of all strokes, 87% are ischemic^[1]^. In Brazil, stroke is one of the leading causes of death. Despite encouraging data regarding age-standardized declining stroke incidence and mortality in Brazil and globally, the aging population and accumulation risk factors contribute to an increased lifetime risk of stroke^[2]^.

Stroke remains a devastating complication after cardiac surgical procedures with substantial functional and economic impacts^[3]^. Despite all efforts, the incidence of postoperative stroke remains as high as 6%^[4]^. Even when patients survive to discharge, they have increased hospital length of stay and are more likely to need to be discharged to a nursing home or a rehabilitation center. Thirty-day mortality is also increased in these patients^[5]^. There are many attributed causes of postoperative stroke. Carotid artery stenosis (CAS) and aortic plaque load are predominantly responsible. However, cardiac emboli, cerebral vasospasm, particulate emboli from cardiopulmonary bypass (CPB), transient hypercoagulable state, and hypoperfusion have also been implicated. The presence of multiple risk factors in the majority of patients makes understanding the exact etiology challenging^[6]^.

Previous studies have attempted to identify predictors of stroke after coronary artery bypass grafting (CABG) with or without combined valvular procedures. These factors seem to be related not only to clinical characteristics (*e.g.*, advanced age, heart failure, diabetes mellitus) but also to the process of care, such as prolonged CPB^[7-10]^. The results are so far conflicting or incomplete mainly due to a low number of interventions or a low number of variables^[11]^. Additionally, Brazil and other Latin-American countries lack contemporary statistics regarding stroke risk after cardiac surgery.

Therefore, the present study aimed to investigate the incidence and predictors of perioperative and seven-day postoperative stroke in a retrospective database from a university hospital.

## METHODS

### Study Design

We conducted a retrospective cohort study of 678 consecutive adult patients who underwent cardiac surgery requiring CPB at the Hospital Universitário de Santa Maria, Brazil, between July 1, 2011, and December 31, 2018. This is a public teaching hospital located in the south of the Brazilian territory that is a reference for approximately 550,000 people. All patients underwent CABG, heart valve surgery alone, or heart valve surgery combined with CABG. The Research Ethics Board at Universidade Federal de Santa Maria (Santa Maria, Rio Grande do Sul, Brazil), approved this protocol, under number CAAE 92068318000005346, and waived the need for individual patient informed consent.

For this analysis, we included a consecutive series of adult patients (age ≥ 18 years) undergoing CABG alone, valvular surgery alone, or a combination of both, all necessitating extracorporeal circulation. The exclusion criteria were surgeries without extracorporeal circulation or any surgical intervention involving carotid or pulmonary arteries, congenital defects, or ventricular aneurysms correction.

All patients received a preoperative evaluation with biochemical analysis, left ventricular function assessment, and carotid ultrasound. Patients who underwent CABG were submitted to a traditional revascularization approach with medial sternotomy, cardioplegic arrest, and CPB support under hypothermia and standardized anticoagulation. After cross-clamp application and inducement of cardioplegia, the anastomoses were performed, including at least one internal mammary artery, if feasible, with the objective of complete revascularization. Once proximal and distal anastomoses were completed, the aorta and grafts were de-aired with subsequent removal of aortic clamp, following myocardial reperfusion. Intraoperative inotropic agents were used as necessary, as well as mechanical circulatory support. Due to limited resources and technical considerations, intraoperative transesophageal echocardiography and off-pump procedures were not performed. For valvular surgeries, the use of mechanical or biological prosthesis was left to the discretion and judgment of the medical team.

Regarding medications, the use of aspirin, beta-blockers, angiotensin-converting enzyme inhibitors or angiotensin-receptor blocking agents, and amiodarone was maintained. P2Y12 receptor inhibitors were withheld for at least five days preoperatively whenever possible. In the postoperative period, systemic anticoagulation was started with unfractionated heparin as indicated as soon as hemostasis was achieved. For those with indication for permanent anticoagulation, vitamin K antagonists were started as soon as possible. For those with transient postoperative atrial fibrillation (POAF), anticoagulation was left as the discretion of the cardiology team^[12]^. Atrial fibrillation (AF) ablation or left atrial appendage occlusion were not performed, since these complex procedures were not available in our medical public system.

All patients had their preoperative, operative, and postoperative information entered in a database using Microsoft Excel® (Microsoft Corporation, United States of America). This database contained all information regarding patients’ demographics, comorbidities, intraoperative management and hemodynamics, postoperative interventions, and hospital outcomes. Due to limited resources and poor long-term follow-up, we were unable to document long-term clinical outcomes after stroke, including death, functional status, or neurological recovery.

The primary outcome was the rate of early (perioperative and seven-day postoperative) stroke, defined as the occurrence of the outcome during the index admission. Stroke was defined as any new permanent focal or global neurologic deficit that appears and is still at least partially evident more than 24 hours after its onset, occurring during or after the cardiac procedure and established before discharge, following published guidelines^[13]^. All new cases of stroke underwent neurologic evaluation and medical imaging with brain computed tomography (CT) or magnetic resonance imaging (MRI) before definitive diagnosis. Routine cerebral scans or neurologic evaluation were not performed in asymptomatic patients.

### Statistical Analysis

Statistical analysis was performed using the IBM Corp. Released 2011, IBM SPSS Statistics for Windows, version 20.0, Armonk, NY: IBM Corp. The Shapiro-Wilk test was used for the determination of quantitative data distribution. Continuous variables with normal distribution were described as mean ± standard deviation. Continuous variables with non-symmetrical distribution were described as median and interquartile ranges. Categorical variables were expressed as percentages. We used Poisson regression analysis with simple error variance to determine risk factors that individually contributed to stroke risk. Risk factors for stroke were examined with the use of univariate analysis; factors with a *P*-value < 0.10 were included in the multivariate analysis. The variables that showed statistical significance were then analyzed through Poisson regression analysis with robust variance. A *P*-value < 0.05 with a confidence interval (CI) of 95% was considered statistically significant.

## RESULTS

The population consisted of 678 patients who underwent cardiac surgery between July 1, 2011, and December 31, 2018. In the overall sample, mean age was 61.4±10.7 years, and men comprised 469 (69.2%) patients. Medical comorbidities include hypertension in 556 (82%), type 2 diabetes mellitus in 240 (35.4%), and coronary artery disease in 522 (77%) patients.

Surgical procedures were distributed as follows: CABG in 461 (68%), aortic valvular replacement in 85 (12.5%), and mitral valve replacement in 44 (6.5%) patients. Table 1 describes the clinical characteristics of the sample and the surgical procedure.

**Table 1:** Epidemiologic characteristics of the population.

Variables	N=678 (%)
Age^*^	61.4±10.7
Female sex	209 (30.8)
Hypertension	556 (82)
DM	240 (35.4)
Current smoker	128 (19.8)
COPD	28 (4.1)
Obesity	147 (22.6)
CAD	522 (77)
Recent MI (< 90 days)	214 (31.6)
Ejection fraction ≤ 40%	63 (9.7)
Ejection fraction (%)^*^	58.3±12.1
Carotid artery disease	216 (41.1)
TIA/stroke	61 (9.1)
TIA/stroke, treatment	
Carotid stent	2 (0.3)
Endarterectomy	1 (0.1)
Clinical treatment	58 (8.7)
PAD	42 (6.2)
Creatinine clearance (ml/min)^*^	71.0±26.4
Hemodialysis	9 (1.3)
Atrial fibrillation	61 (9.0)
Infective endocarditis	25 (3.7)
Symptoms on admission	
Stable angina	177 (26.2)
Unstable angina	113 (16.7)
MI	178 (26.3)
Other	161 (23.8)
No symptoms	47 (7.0)
Acute decompensated heart failure	313 (46.3)
Previous cardiac surgery	29 (4.3)
Urgent surgery	361 (53.3)
EuroSCORE	2.0% (1.2%; 4.0%)
Preoperative cardiogenic shock	13 (1.9)
Intra-aortic balloon pump	8 (1.2)
Time waiting surgery (days)†	7.0 (1.12)
Type of procedure	
CABG	461 (68.0)
CABG + AVR	39 (5.7)
CABG + MVR	18 (2.6)
AVR	85 (12.5)
MVR	44 (6.5)
AVR + MVR	15 (2.2)
Other procedures	16 (2.4)
Extracorporeal circulation ≥ 110 min	224 (33.8)
Time in extracorporeal circulation^*^	102.7±35.6
Internal mammary artery graft	484 (71.4)
Venous graft (number)	
0	39 (5.7)
1	100 (14.7)
2	241 (35.5)
≥ 3	137 (20.2)
Valve type	
Mechanical	76 (11.2)
Biological	128 (18.9)

AVR=aortic valve replacement; CABG=coronary artery bypass grafting; CAD=coronary artery disease; COPD=chronic obstructive pulmonary disease; DM=diabetes mellitus; EuroSCORE=European System for Cardiac Operative Risk Evaluation; MI=myocardial infarction; MVR=mitral valve replacement; PAD=peripheral arterial disease; TIA=transient ischemic attack

* Mean ± standard deviation

†Median and interquartile range

Overall mortality in the population was 47 (7.1%) cases. The main cause of death in the population was cardiogenic shock (3.4%), followed by sepsis (2.1%). Nineteen (2.8%) patients required surgical reintervention within 30 days. The main reasons for reinterventions were bleeding (1.1%), mediastinitis (0.4%), and cardiac tamponade (0.4%). The mean length of hospital stay was 16 days (interquartile range 11-25), with intensive care unit (ICU) stay of five days (interquartile range 4-7). Thirteen patients (2%) were readmitted to the ICU.

The incidence of postoperative infection was 209 (31.1%) cases, with the following distribution: pulmonary infection in 116 (17.1%) and surgical wound infection in 49 (7.2%) patients. Postoperative stroke occurred in 24 patients (3.5%), 23 (3.3%) were ischemic, and 21 (3.0%) were diagnosed in the first 72 hours after surgical procedure. Two individuals had a presumptive diagnosis of stroke after neurological evaluation but were excluded from the sample because they did not have a complementary imaging exam. The other outcomes are shown in Table 2.

**Table 2:** Postoperative outcomes.

Variables	Total (%) N=678
Postoperative stroke	24 (3.5)
Ischemic	23 (3.3)
24 hours to < 72 hours	21 (3.0)
≥ 72 hours	3 (0.4)
Postoperative infarction	81 (12.1)
Reintubation	30 (4.6)
Atrioventricular block	25 (3.7)
Acute atrial fibrillation	215 (31.8)
Use of vasoactive/inotropic drugs	627 (93.9)
< 24 hours	499 (74.7)
≥ 24 hours and < 72 hours	91 (13.6)
≥ 72 hours	37 (5.5)
Infection	209 (31.1)
Pulmonary	116 (17.1)
Surgical wound	49 (7.2)
Urinary	16 (2.4)
Bloodstream/catheter	4 (0.6)
Other	7 (1.0)
Surgical reintervention	19 (2.8)
Surgical reintervention, causes	
Increased bleeding	7 (1.1)
Mediastinitis	3 (0.4)
Cardiac tamponade	3 (0.4)
Sternum dehiscence	1 (0.2)
Retrosternal hematoma	1 (0.1)
Constrictive pericarditis	1 (0.2)
Pericardium clot	1 (0.1)
Rupture of the right ventricle	1 (0.1)
Valvular leak	1 (0.1)
ICU stay, median (IQR)	5 (4 - 7)
ICU readmission	13 (2)
Mechanical ventilation time	
< 24 hours	617 (92)
≥ 24 hours and < 72 hours	26 (3.8)
≥ 72 hours	28 (4.2)
Tracheostomy	9 (1.3)
Hemodialysis	15 (2.2)
Death	48 (7.0)
Causes of death	
Cardiogenic shock	23 (3.5)
Septicemia	14 (2.1)
Mixed shock	5 (0.7)
DIVC/coagulopathy	2 (0.3)
Hemorrhagic shock	1 (0.1)
Pulmonary embolism	1 (0.1)
Stroke	1 (0.1)
Subdural hematoma	1 (0.1)
Hospital stay, median (IQR)	16 (11 - 25)

DIVC=disseminated intravascular coagulation; ICU=intensive care unit; IQR=interquartile range

The univariate predictors of seven-day ischemic stroke were determined in Table 3. After multivariate analysis, the following factors were significantly associated with stroke: previous stroke/transient ischemic attack (TIA) (relative risk [RR]=2.75; 95% CI, 1.11-6.82; *P*=0.029), carotid artery disease (RR=4.0; 95% CI, 1.43-11.0; *P*=0.008), previous AF (RR=3.26; 95% CI, 1.31-8.1; *P*=0.011), and postoperative platelets > 200,000/mm^3^ (RR=2.26; 95% CI, 1.01-5.1; *P*=0.048) (Table 4).

**Table 3:** Risk factors for seven-day stroke in univariate analysis.

Variables	No stroke N=654 (96.5%)	Stroke N=24 (3.5%)	RR	95% CI	*P*-value
Age^*^	61.4±10.8	62.5±97	1.0	0.975 - 1.045	0.607
Female	202 (30.9)	7 (29.1)	0.9	0.389 - 2.194	0.858
SAH	537 (82.1)	19 (79.2)	0.8	0.318 - 2.190	0.712
DM	228 (34.9)	12 (50)	1.8	0.833 - 3.999	0.133
Obesity	141 (21.6)	6 (25)	1.3	0.511 - 3.220	0.595
Active smoking	125 (19.1)	3 (12.5)	0.5	0.143 - 1.777	0.287
COPD	26 (4.0)	2 (8.3)	2.1	0.522 - 8.534	0.295
Ischemic heart disease	503 (76.9)	19 (79.1)	1.1	0.431 - 2.992	0.797
Recent heart attack (< 90 days)	207 (31.6)	7 (29.2)	0.9	0.394 - 2.257	0.894
Ejection fraction ≤ 40%	69 (10.5)	3 (12.5)	1.1	0.349 - 3.730	0.827
Ejection fraction^*^	58.3±12.1	57.6±12.4	1.0	0.965 - 1.027	0.780
Carotid disease	201 (30.7)	15 (62.5)	4.3	1.594 - 11.708	0.004
Previous TIA/stroke	55 (8.4)	6 (25)	3.3	1.377 - 8.095	0.008
PAD	39 (6.0)	3 (12.5)	2.2	0.672 - 6.961	0.196
Creatinine clearance^*^	71.2±26.7	67.3±18.5	1.0	0.983 - 1.006	0.340
Endocarditis	23 (3.5)	2 (8.3)	2.4	0.591 - 9.546	0.223
Previous atrial fibrillation	56 (8.6)	5 (20.8)	2.6	1.027 - 6.856	0.044
Decompensated CHF	301 (46.0)	12 (50.0)	1.2	0.529 - 2.545	0.712
Preoperative hemoglobin	13.0±1.8	12.6±1.8	0.9	0.732 - 1.085	0.250
Preoperative platelets (×10^3^/mm^3^)^*^	224.3±69.6	266.4±100.3	1.1	1.021 - 1.105	0.003
Preoperative INR	1.1 (1.0; 1.1)	1.1 (1.1; 1.2)	3.7	0.958 - 14.403	0.058
Previous cardiac surgery	27 (4.1)	2 (8.3)	2.0	0,502 - 8.241	0.320
Emergency surgery	347 (53.1)	14 (58.3)	1.2	0,552 - 2.720	0.617
Procedure type					
MRS	445 (68.0)	16 (66.7)	1.0	-	-
Valve exchange	132 (20.2)	2 (8.3)	0.4	0.100 - 1.847	0.114
Combined procedure	77 (11.8)	6 (25)	2.1	0.839 - 5.168	0.256
ECC time ≥ 110 min	215 (32.9)	9 (37.5)	1.3	0.553 - 2.859	0.585
CPB time^*^	102.7±35.3	108.8±38.7	1.0	0.995 - 1.014	0.393
Cardiogenic shock	107 (16.3)	6 (25)	1.7	0.677 - 4.106	0.267
CRP/ventricular arrhythmias	68 (10.4)	4 (16.7)	1.7	0.592 - 4.788	0.329
Acute atrial fibrillation	207 (31.6)	8 (33.3)	1.1	0.467 - 2.472	0.866
Postoperative fibrinogen^*^	260.2±102.3	264.6±101.5	1.0	0.997 - 1.004	0.831

* mean ± standard deviation

CHF=congestive heart failure; CI=confidence interval; COPD=chronic obstructive pulmonary disease; CPB=cardiopulmonary bypass; CRP=cardiorespiratory arrest; DM=diabetes mellitus; ECC=extracorporeal circulation; INR=international normalized ratio; MRS=myocardial revascularization surgery; PAD=peripheral arterial disease; RR=relative risk; SAH=systemic arterial hypertension; TIA=transient ischemic attack

**Table 4:** Risk factors for seven-day stroke in multivariate analysis.

Variable	Relative risk	95% CI	Significance
Previous stroke/TIA	2.75	1.11 - 6.82	0.029
Carotid artery disease	4.0	1.43 - 11.0	0.008
Previous atrial fibrillation	3.26	1.31 - 8.1	0.011
Platelets > 200,000/mm^3^	2.26	1.01 - 5.1	0.048

CI=confidence interval; TIA=transient ischemic attack

## DISCUSSION

Neurologic complications after cardiac surgical procedures remain relatively common despite improvements in anesthetic and surgical techniques, as well as in perioperative monitoring and management^[14]^. Previous studies have demonstrated that neurologic deficits occur in as many as 6% of patients undergoing cardiac surgery^[5,9,15,16]^. However, in a contemporary metanalysis, Gaudino et al.^[14]^ described a pooled event rate of perioperative stroke of 2.03%, with an incidence of ischemic stroke of 88%. The incidence of clinically silent cerebral infarction though is higher (up to 18%), particularly after aortic valve replacement surgery^[4]^. The occurrence of early stroke is associated with a significant increase in mortality as well as much higher increase in the risk of late death, suggesting that addressing this potential complication can significantly improve the outcomes of cardiac surgery^[17]^.

In this single-center study, we determined predictors of early (perioperative and seven-day postoperative) stroke with CABG, heart valve surgery alone, or heart valve surgery combined with CABG. We observed a stroke rate of 3.5% (24 patients), with 3.3% (23 patients) suffering an ischemic event. Additionally, 3.0% of the strokes (21 cases) were diagnosed in the first 72 hours after the surgical procedure. These numbers are high compared to the contemporary series of 2.3% of perioperative ischemic strokes^[14]^. In the last decades, significant efforts have been aimed at perioperative stroke reduction, including minimizing aortic manipulation, eliminating CPB, and using preoperative CT scan of the ascending aorta or duplex scanning of the carotid arteries as well as epiaortic ultrasound^[18-20]^. We reported a higher number of such complication, and the reasons may be related to the lack of these strategies in our service, which suggest that early stroke is probably linked to intraoperative events, reflecting the technical or surgical nature of its etiology^[14,17,21]^. We also must acknowledge that the present series represents data of a single cardiology center with only two surgeons available and low annual surgery rate. As previously reported, increased surgeon and hospital volume significantly improves outcomes in cardiovascular surgery, although the benefit is less pronounced than in other specialties^[22]^.

After multivariate analysis, we determined the following independent factors associated with stroke: previous stroke or TIA^[23]^, carotid artery disease, previous AF, and early postoperative platelet counts > 200,000/mm^3^. In the present series, we demonstrated that a history of previous stroke or TIA was an independent risk factor for the development of postoperative stroke, increasing the risk of the event by 2.75. Similar findings were reported by Hogue et al.^[16]^. Additionally, the documentation of significant carotid artery disease, with stenosis > 50% in internal carotid arteries, was associated with four times the risk of stroke. In a Brazilian series by Costa et al.^[24]^, the rate of ischemic stroke in patients with carotid stenosis ≥ 50% was 10%, while in patients with carotid stenosis ≥ 70% this index reached 16.6%. It is suggested that up to 30% of early postoperative strokes complicating CABG are caused by a hemodynamically significant CAS; however, some evidence disputes this presumption. Contemporary data suggest that the single most significant marker of an adverse cerebral outcome after CABG is the identification of atherosclerotic plaques in the ascending aorta, suggesting carotid lesions are more a risk marker than the etiology of stroke. The high prevalence of aortic atherosclerosis in patients with CAS may help to explain the higher stroke rate in patients with lesions outside the territory of their carotid disease. This also creates great doubt about the benefits of prophylactic treatment of carotid lesions in patients without neurological symptoms^[25]^.

Regarding arrhythmia, the presence of AF was an independent predictor of stroke in our study, with an RR of 3.26. The embolic mechanism associated with clots in the left atrium is the most probable explanation for this association, particularly in patients with unsatisfactory control of anticoagulation or intraoperative surgical manipulation or spontaneous recovery of sinus rhythm postoperatively^[26]^. Pierik et al.^[27]^ performed a secondary analysis from a randomized trial and observed that patients with POAF within the first seven days after cardiac surgery had a three-fold increased risk for thromboembolic stroke during hospital admission based on the difference in the incidence of POAF between patients with and without thromboembolic stroke. In a meta-analysis published by Kaw et al.^[28]^ and Lin et al.^[29]^, the development of new-onset POAF after cardiac and non-cardiac surgery was associated with increased shortand long-term stroke and mortality. Several series have confirmed a higher long-term mortality after the development of POAF in cardiac surgery^[30-32]^. There are published guidelines to support the prevention of AF in cardiac surgical patients, but there is insufficient evidence to recommend rhythm control for stroke prevention once fibrillation has occurred^[33]^.

Finally, our study found postoperative platelet counts > 200,000/mm^3^ as an independent risk factor for stroke. This finding is somehow surprising and opposite to previous evidence. Karhausen et al.^[34]^ demonstrated in a single-center study that moderate to severe postoperative thrombocytopenia was an independent risk factor for the development of postoperative stroke. Griffin et al.^[35]^ showed that postoperative thrombocytopenia is independently associated with postoperative mortality, acute kidney injury, infection, stroke, and prolonged ICU and hospital lengths of stay. The mechanism for developing thrombocytopenia in these patients remains unclear, but the association of postoperative platelet counts with stroke after CABG surgery suggests that the reduction in platelet numbers likely occurs in a context of increased platelet reactivity^[34]^.

### Limitations

Several limitations involved in this study should be noted. First, it is limited by its dependence on a retrospective observational design, and the conclusions derived from this study are necessarily limited in application.

Second, it is a single-center study with a relatively small sample and limited number of events. Therefore, one must acknowledge the study is underpowered. Probably because of the small number of events, we were unable to show classic predictors of perioperative stroke, like advanced age, female sex, peripheral vascular disease, combined CABG and valve surgery, preoperative infection, new-onset AF, or high transfusion requirements^[9,36]^. Regarding patients who developed POAF, the dataset probably captured the instances only that this arrhythmia was clinically significant and prolonged enough to be registered. There was also no specific registration regarding time of anticoagulation initiation, neither the precise timing of stroke in relation to the onset of POAF.

Third, the stroke diagnosis was strictly based on neurological evaluation annexed with postoperative CT or MRI; the severity of each stroke and each patient’s functional status remains unknown and cannot be compared.

Despite these limitations, our study includes a significant sample of cases in the south of the Brazilian territory and reinforces some classic risk factors for postoperative stroke after cardiac surgery. Additionally, most models found in the literature describe the risk of stroke after isolated CABG, with no stroke risk determination after combination (CABG with valve surgery or valvular surgery alone).

Larger prospective studies with long-term follow-up are necessary to address prognostic markers for stroke and mortality.

## CONCLUSION

We demonstrated that a history of previous stroke or TIA, carotid artery disease, previous AF, and platelet counts > 200,000/mm^3^ showed as independent risk factors for the development of postoperative stroke.

**Table:** 

Authors’ Roles & Responsibilities	
LQM	Substantial contributions to the conception or design of the work; drafting the work or revising it critically for important intellectual content; final approval of the version to be published
MALS	Substantial contributions to the conception or design of the work; drafting the work or revising it critically for important intellectual content; final approval of the version to be published
LFS	Substantial contributions to the conception or design of the work; drafting the work or revising it critically for important intellectual content; final approval of the version to be published
MCML	Substantial contributions to the conception or design of the work; drafting the work or revising it critically for important intellectual content; final approval of the version to be published
PCA	Substantial contributions to the conception or design of the work; and the analysis or interpretation of data for the work; drafting the work or revising it critically for important intellectual content; final approval of the version to be published
DC	Substantial contributions to the conception or design of the work; and the analysis or interpretation of data for the work; drafting the work or revising it critically for important intellectual content; final approval of the version to be published
